# Cleaning the Teeth

**Published:** 1897-06

**Authors:** J. C. Wing

**Affiliations:** England.


ARTICLE V.

### CLEANING THE TEETH.

BY DR. J. C. WING, ENGLAND.

An ordinary simple tooth powder should contain:

A scouring and polishing powder, of fine quality. such as precipitated chalk. An antiseptic. An antiacid, such as carbonate of magnesia. A cleansing agent in the form of a neutral soap. A flavoring agent, such as attar of roses, or wintergreen.

Advertised powders and washes should be avoided, as the ingredients are not known. Any dentifrice that is recommended for whitening the teeth is either incapable of accomplishing what is claimed for it, or does so at the expense of the integrity of the enamel. It is a good plan, in acid saliva, to rub a little precipitated chalk into the interstices of the teeth the last thing at night. The quantity need only be sufficient to counteract the acidity during the night. Lime water may be used as a mouth-wash where the mucous secretions are viscid or fetid, and it also has the effect of increasing the lime in the structure of the teeth. Its unpleasant taste may be disguised by a flavor-agent.

Phenol-sodique makes a good mouth-wash, as it is anti-septic, astringent, antacid, styptic and disinfectant. Listerine, izal, and other preparations of a like nature are also useful. More than usual care should be taken during sickness and pregnancy to see that the mouth is kept in a clean aseptic condition, this consideration being frequently overlooked.—*British Journal.*

